# Fertilization can accelerate the pace of soil microbial community response to rest‐grazing duration in the three‐river source region of China

**DOI:** 10.1002/ece3.10734

**Published:** 2023-11-27

**Authors:** Xuanbo Zhou, Xiaoli Wang, Yushou Ma, Yanlong Wang, Yuan Ma, Lele Xie

**Affiliations:** ^1^ Academy of Animal Husbandry and Veterinary Sciences, Key Laboratory of Alpine Grassland Ecosystem in the Three‐River‐Source (Qinghai University), Ministry of Education, Qinghai Provincial Key Laboratory of Adaptive Management on Alpine Grassland Qinghai University Xining Qinghai China

**Keywords:** fertilization, rest‐grazing during the regreen‐up period, soil microbial community, three‐river source region

## Abstract

Overgrazing leads to grassland degradation and productivity decline. Rest‐grazing during the regreen‐up period can quickly restore grassland and fertilization is a common restoration strategy. However, the effects of rest‐grazing time and fertilization on soil microorganisms are unclear in the alpine grasslands. Therefore, the experiment of rest‐grazing time and fertilization was carried out to explore the response of soil microorganisms to rest‐grazing time and fertilization measures. A field control experiment with rest‐grazing time and fertilization as factors have been conducted from the time when grass returned to green till the livestock moved to the summer pasture in Dawu Town of Maqin County of China. The primary treatment we established was the five rest‐grazing time, including rest‐grazing time of 20 days, 30 days, 40 days, 50 days, and traditional grazing was used as a check group. At the same time, the secondary treatment was nitrogen addition of 300 kg·hm^−2^ in each primary treatment. The results showed that the total phospholipid fatty acid (total PLFA), actinomyces (Act), and arbuscular mycorrhizal fungi (AMF) showed an ever‐increasing biomass with the increase of rest‐grazing time and the highest was at 50 days of rest‐grazing, and they were all significantly higher than CK. In addition, soil microbial biomass carbon‐nitrogen ratio (MBC/MBN) had great influence on the change of microbial community. Applying nitrogen fertilizer can increase the maximum value of biomass of all PLFA groups and the biomass of all PLFA groups changed in an “inverted V” shape with the increase of rest‐grazing time. Besides, instead of MBC/MBN, NO_3_^−^‐N was positively correlated with the biomass of all PLFA groups, which actively regulated the trend of microbial functions. The longer rest‐grazing time is more conducive to the biomass of all PLFA groups. However, applying nitrogen fertilizer could break this pattern, namely, the 30 days rest‐grazing would be beneficial to the biomass of all PLFA groups. These findings provide key information that rest‐grazing during the regreen‐up period is benefiscial to the all PLFA groups and fertilization could change the response of microorganisms to rest‐grazing, which provide reference measures for the restoration of degraded alpine meadows.

## Graphical abstract

The longer rest‐grazing time is more conducive to the biomass of all PLFA groups. However, applying nitrogen fertilizer could break this pattern, namely, the 30 days rest‐grazing would be beneficial to the biomass of all PLFA groups. These findings provide key information that rest‐grazing during the regreen‐up period is beneficial to the all PLFA groups and fertilization could change the response of microorganisms to rest‐grazing, which provide reference measures for the restoration of degraded alpine meadows.

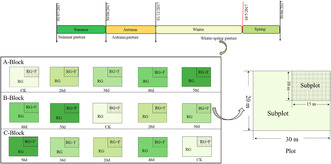

## 1 INTRODUCTION

Grasslands are under severe threat from ongoing degradation, undermining their capacity to support biodiversity, ecosystem services, and human well‐being. (Bardgett et al., 2021). The three‐river source region of China located in the Qinghai‐Tibet Plateau, is not only an important eco‐security barrier in China, but also an area with highly sensitive and vulnerable natural eco‐systems (Sun et al., 2023). Alpine meadow is the most widely distributed vegetation type in the three‐river source region of China, and is an important contributor to maintaining grassland biodiversity but also the material basis for the development of animal husbandry (Zhang et al., 2016). With the influence of climate change and human factors, the degradation of the ecological environment in the three‐river source region has attracted the attention of many scholars (Zhou et al., 2021). The degradation is a process of retrogressive succession of grasslands, which is mainly manifested by the changes in the dominance of good pastures decline, biodiversity loss, reduction of grassland productivity, and deterioration of soil habitat (Lyu et al., 2022). Therefore, in the process of grassland degradation, soil physicochemical properties may be altered prominently, which can influence the soil microbial community, and further influence ecosystem functions and services (Zhang et al., 2023).

Alpine meadow ecosystems are fragile, unstable and difficult to self‐repair, and grasslands are degraded under the disturbance of natural and human factors (Li, Zhang, et al., 2023). Grazing impacts on ecosystem services remain uncertain because pervasive interactions between grazing pressure, climate, soil properties, and biodiversity may occur but have never been addressed simultaneously (Maestre et al., 2022). However, overgrazing is the main cause of grassland degradation in human factors (Wang, Wang, et al., 2021). Therefore, it is necessary to optimize the grazing system, especially the critical period of the regreen‐up, to alleviate the degradation of alpine meadow (Sun et al., 2020). At present, grazing prohibition (Wang, Weng, et al., 2022), rotational grazing (Zander et al., 2019), fence enclosure (Xu et al., 2020), and rest‐grazing during the regreen‐up period have been carried out (Zhou et al., 2022). The regreen‐up period of forage grass is the most vulnerable period of forage grass. The photosynthetic area of newly germinated forage grass will decrease rapidly after being eaten, which directly affects its growth and development in the later stage (Ma et al., 2022). Therefore, the pasture regreen‐up period is the “prevention period” for natural grassland in the three‐river source region (Ma et al., 2017). Many studies in temperate steppes of Inner Mongolia and alpine meadows of the Qinghai‐Tibet Plateau have shown that banning and rest‐grazing during the regreen‐up period has effectively promoted plant height, vegetation coverage, species richness, and soil restoration (Cai et al., 2022; Li et al., 2014, 2017). However, the rest‐grazing policy has been implemented for many years, but it is still unclear how long is it better to rest grazing during the regreen‐up period. The initial rest‐grazing period and the duration of rest‐grazing are the key issues to determine the scientific rest‐grazing. At the same time, livestock need to be housed and fed with concentrated feed during the rest‐grazing period. Moreover, a too‐long rest‐grazing period may reduce the forage supply for livestock, which can significantly increase the cost of feeding (Briske et al., 2015). In order to give consideration to both economic benefits and ecological benefits, it is necessary to choose an appropriate date to start rest‐grazing, so as to protect the grassland from being disturbed by grazing during the regreen‐up period, and also effectively avoid hindering normal grazing activities for a long time.

At the same time, the degraded grassland has led to a decrease of grassland productivity and also led to soil degradation, resulting in a gradual decrease in soil fertility (Li et al., 2022). Soil degradation caused by grazing is an emerging global problem that threatens soil communities and ecosystem functioning (Zhou et al., 2023). In a short period of time, it is difficult to restore the fertility quickly, so fertilization is needed to restore the soil fertility quickly (Teng et al., 2020). Meanwhile, nitrogen fertilizer application can cause changes in soil microbial activity and soil microbial community structure by affecting the chemical composition of the soil but also can affect the growth of aboveground vegetation by changing the physicochemical properties of the soil, thereby indirectly affecting the structure of the soil microbial community (Sun et al., 2018; Wang, Zhang, et al., 2022). Soil microbial are known to contribute to soil nutrient mobilization and turnover (Xu et al., 2023a). Therefore, applying nitrogen fertilizer on degraded alpine meadow is beneficial to the rapid recovery of soil fertility, and then to the recovery of aboveground vegetation and soil microorganisms.

Changing microbial communities can alleviate environmental stress and promote plant growth (Xu et al., 2023b). Soil microorganisms are an important part of the grassland ecosystem, and play an irreplaceable role in regulating plant growth, promoting the formation of soil structure, and maintaining the function and stability of the grassland ecosystem (Brown et al., 2022). Therefore, some studies have shown the responses of soil microbial community to grassland degradation (Chao et al., 2022; Wang, Wen, et al., 2020). To study microbial diversity in complex soil communities, phospholipids fatty acid (PLFA) biomarker methods are widely used in ecological research (Norris et al., 2023). Due to its structural diversity and biological specificity, PLFA is a particularly effective biomarker and can be used to understand the structure of microbial communities (Barreiro et al., 2022). In recent years, research on soil microbial diversity and community structure changes has mainly focused on different rest‐grazing measures, while there were few reports about rest‐grazing time on alpine meadows in the three‐river source region (Wang, Ren, et al., 2020; Zhao et al., 2023).

In this study, we hypothesized that rest‐grazing during the regreen‐up period can alter the soil microbial community structure, and the longer the rest‐grazing, the higher the biomass of soil microbial groups. Furthermore, the fertilization could increase the biomass of microorganisms and change the response of soil microorganisms to rest‐grazing in the degraded grassland. Our objective was clear of the effects of rest‐grazing and fertilization during the regreen‐up period on soil microbial diversity and community structure. And the experiment provided references for adopting more appropriate policies of rest‐grazing and fertilization. Moreover, it can promote regional ecological conservation of grasslands and sustainable development of animal husbandry. To do this, the experiment was carried out in the three‐river source region of China, which added the treatment of applying nitrogen fertilizer under the premise of rest‐grazing during the regreen‐up period.

## 2 MATERIALS AND METHODS

### 2.1 Study area

This study was conducted at Yongbao Animal Husbandry Committee, Dawu Town, Guoluo Tibetan Autonomous Prefecture (34°24′14″ N, 100°23′31″ E), with an altitude of 3920 m. It belongs to the alpine climate of the Qinghai‐Tibet Plateau (Mao et al., 2021). The average annual temperature of −3.9°C, so that the temperature is relatively low. And there is large temperature difference between day and night. The annual precipitation between 423 and 565 mm, and the annual evaporation is 2471.6 mm (Ning et al., 2022). There is no absolute frost‐free period throughout the year, and the grass growing season is about 156 days (Ma et al., 2007). The soil type is alpine meadow soil and the grassland type is alpine meadow (Xu et al., 2013). The natural vegetation mainly consists of *Poa pratensis*, *Elymus nutants*, *Kobresia pygmaea*, *Scirpus distigmaticus*, *Ajania tenuifolia*, and *Pleurospermum hookeri* as the dominant species.

### 2.2 Experimental design

**FIGURE 1:**
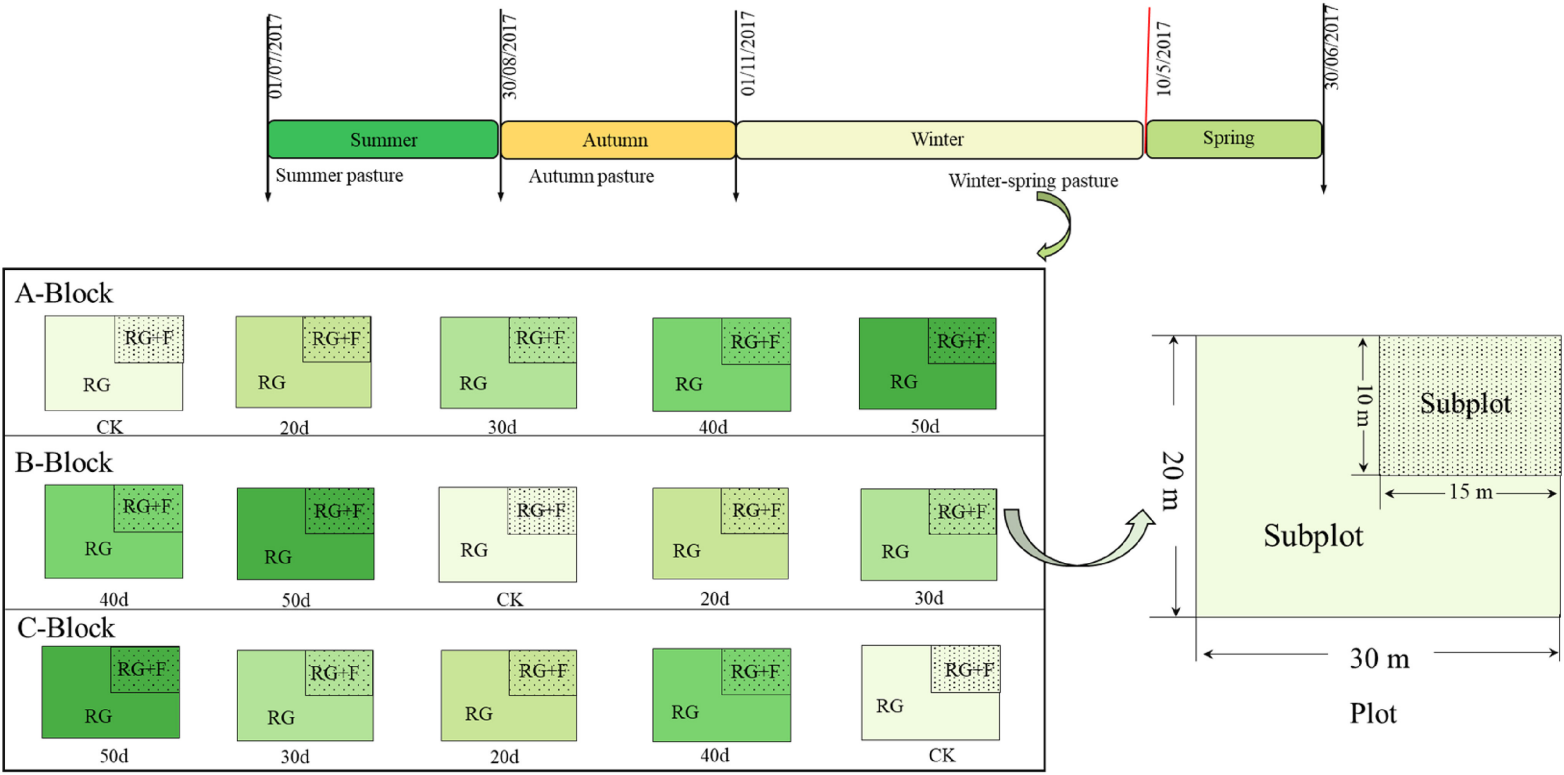
Layout of the field control experiment. *Note*: Winter–spring pasture was grazing in winter and spring but rest‐grazing in summer and autumn. A‐Block, B‐Block, and C‐Block: three independent replicate blocks; CK, control check group; 20d, rest‐grazing from June 10 to June 30; 30d, rest‐grazing from May 30 to June 30; 40d, rest‐grazing from May 20 to June 30; 50d, rest‐grazing May 10 to June 30; RG, rest‐grazing treatment; RG + F, rest‐grazing with fertilization treatment; there were 2 subplots in each plot and there were 5 plots in each block.

The winter–spring pasture with the mild degradation grassland degradation was selected as the experimental site, covering an area of 2 ha. In recent 30 years, the experimental site had been used as a winter–spring pasture, and it was rest‐grazing in summer and autumn. When the experiment began, we tried to choose the grassland with consistent site conditions. The primary treatment we established was the five rest‐grazing time, including grazing during the whole regreen‐up period as a control check group (CK), rest‐grazing from June 10 to June 30 (20d), rest‐grazing from May 30 to June 30 (30d), rest‐grazing from May 20 to June 30 (40d), and rest‐grazing from May 10 to June 30 (50d). The rest rates of regreen‐up period in each treatment were 0, 40%, 60%, 80%, and 100%, respectively. In this study the grazing intensity was moderate and light grazing, and the grazing intensity is about 0.89–1.45 cattle ha. At the same time, the secondary treatment we established was fertilization in each primary treatment (urea N content was 46%). According to the fertilization amount of alpine meadow grassland type and previous researches, the final fertilization amount was 300 kg·hm^−2^ (Jing et al., 2014; Li et al., 2004; Ma et al., 2003). On June 20th, 2017, nitrogen fertilizer was applied once. We established three independent replicate blocks (A, B, and C) and five plots (CK, 20d, 30d, 40d, and 50d) of 30 × 20 m were randomly set up in each block. In each plot, a quarter subplot (15 m × 10 m) of the northeast corner of each plot as the fertilization treatment. In total, we had set up 15 experimental plots and each plot was 20 m apart. Each plot was partitioned into two subplots, 1 subplot was rest‐grazing treatment (RG), and the other was rest‐grazing with fertilization treatment (RG + F). Only the difference of feeding was between the control check and the rest‐grazing treatment, and other factors were the same (Figure 1).

### 2.3 Soil sampling

Soil samples were obtained in August 2017, when the grass was growing vigorously. In each subplot, three soil samples were collected to reduce spatial heterogeneity. And five soil cores (3.5 cm diameter) were taken at a depth of 0–15 cm regarding as one soil sample. After the soil samples were measured, the data of three soil samples from each subplot were averaged to obtain one value. Therefore, only one value was obtained for each subplot. In total, three independent samples were obtained from each treatment of three replicate blocks (A, B, and C). In order to avoid the influence of livestock waste on soil characteristics, we absolutely avoid livestock waste and the traces of livestock waste when sampling. A total of 90 soil samples (three soil samples × 10 subplots × three replicate blocks) were collected in this study. Then, three cutting ring soil samples were collected from a 0–15 cm depths in each plot to determine the soil moisture content (SM). In addition, when collecting soil samples, we remove debris such as plant litter, stone particles and livestock excreta. Each soil sample was sieved through a 2 mm mesh and then divided into two parts when the soil samples were taken back to the laboratory (Joshi & Garkoti, 2023). One part of the fresh soil from the field was stored at 4°C for microbial biomass (carbon and nitrogen), inorganic N (NH_4_^+^−N and NO_3_^−^−N) and available phosphorus (AP) analyses, and the other part was air‐dried indoors and used to determine indicators such as soil physicochemical properties (Zhao et al., 2018).

### 2.4 Analysis of soil properties

Soil samples were collected in a steel ring (Volume 100 cm^3^) for soil SM determination. The soil moisture (SM) content was determined by oven drying the soil samples at 105°C to constant mass. And soil pH was measured by acidity meter (Metter Toledo) ( An et al., 2018). Soil organic carbon (SOC) was determined by K_2_Cr_2_O_7_ oxidation and FeSO_4_ titration methods. Total nitrogen (TN) was determined using Clever Cherm after digestion with H_2_SO_4_‐H_2_O_2_. Inorganic N (NH_4_^+^−N and NO_3_^−^−N) was extracted by potassium chloride. Total phosphorus (TP) was determined by molybdenum antimony anti‐colorimetric method. Determination of available phosphorus (AP) by sodium bicarbonate extraction‐molybdenum antimony anti‐colorimetric method. And total potassium (TK) by flame photometer method (Huang et al., 2014). At last, determination of soil microbial biomass carbon and nitrogen (MNC, MBN) by chloroform fumigation extraction (Brookes et al., 1985).

### 2.5 Determination of phospholipid fatty acids

In order to reveal the effects of rest‐grazing and fertilization during the regreen‐up period on the structure of soil microbial community. Soil microbial PLFA was determined based on the method of Bossio and Scow (1998). Eight grams of each freeze‐dried soil sample was accurately weighed into a 35 mL centrifuge tube, using chloroform‐methanol‐phosphate buffer (volumes were 6 mL, 12 mL and 5 mL in turn) to extract soil microbial PLFA. The tubes were shaken for 2 h and centrifuged at 3000 × *g*. And the supernatant liquid transferred to a 50‐mL separatory funnel. The pellet was re‐extracted and centrifuged as above. Chloroform (12 mL) and phosphate (12 mL) were added to the combined supernatants in the separating funnel. The mixture was shaken briefly and the phases were allowed to separate overnight at room temperature after release of pressure. Collected the lower layer solution of the separatory funnel and then the solvent was evaporated under a stream of oxygen‐free N_2_. Lipids and glycolipids were eluted with silica column (Diameter of Inner/Outer Tube, CNWBOND Si SPE Cartridge, 500 mg, 3 mL). And the eluents were chloroform (2 × 2.5 mL) and acetone (2 × 5 mL), respectively. Polar lipids were eluted with 2 × 2.5 mL methanol and collected in glass test tubes. The solvent was evaporated under a stream of oxygen‐free N_2_ and the residue subjected to alkaline methanolysis by shaking in a mixture (2 mL) of KOH (0.2 mol L^−1^, 1 mL) and toluene: methanol (1:1(v/v), 1 mL), followed by incubation at 37°C for 15 min. After cooling, the mixtures were neutralized with acetic acid (1 mol L^−1^) and fatty acid methyl esters (FAMEs) extracted twice into 2 mL hexane. After evaporation of the solvent under oxygen‐free N_2_, the FAMEs were resuspended in hexane (200 μL) containing 5 μg nonadecanoic acid methyl ester as an internal standard. Identification and quantification of fatty acid methyl ester biomarkers were done on a gas chromatograph (Agilent 7890B) with an Agilent 19091B column. And the extracts were analyzed with the MIDI software system (MgIDI, Inc.). The concentration of each PLFA component was calculated based on 19:0 methyl internal standard concentrations. PLFA was quantified by peak area and internal standard curve method, and PLFA content was expressed by nmol·g^−1^ dry soil. In the classic and recent paper, 16:1ω5c value was used as a biomarker for arbuscular mycorrhizal fungi (Dong et al., 2023; Frostegård & Bååth, 1996). The 16:1ω5c fatty acid was widely regarded as the symbolic fatty acid of arbuscular mycorrhizal fungi, and its content was often used to estimate the biomass and mycelium of AMF (Bossio & Scow, 1998). Therefore, in this paper, 16:1ω5c value was only used as a biomarker for arbuscular mycorrhizal fungi instead of bacteria. The 10Me‐16:0, 10Me‐17:0, 10Me‐18:0 values were used as biomarkers for actinomycete PLFAs (Kroppenstedt, 1985). The values of 18:2ω6, 9c, 18:1ω9c as fungal PLFA biomarkers (F; Frostegård et al., 1993). The i14:0, i15:0, a15:0, i16:0, a17:0, i17:0 values were used as gram‐positive bacterial PLFA biomarkers (G+) and the16:1ω7c, cy‐17:0, 18:1ω5c, 18:1ω7c, cy‐19:0 values were used as gram‐negative bacterial PLFA biomarkers (G−). The14:0, i14:0, 15:0, i15:0, a15:0, 16:0, i16:0, 16:1ω7c,17:0, a17:0, i17:0, cy‐17:0, 18:0, 18:1ω5c, 18:1ω7c, cy‐19:0values were used as total bacterial PLFA biomarkers (B; White et al., 1979). Additionally, the F/B ratio (the ratio of all the fungal PLFA biomarkers to all the bacterial PLFA biomarker biomass ratios) and the G+/G− ratio (the ratio of gram‐positive bacterial PLFA biomass to gram‐negative bacterial PLFA biomass) were also calculated (Sadeghi et al., 2023). The bacterial stress indicators were also calculated as follows: the cy/pre ratio was represented by the ratio of (cy‐17:0 + cy‐19:0) to (16:1 ω7c + 18:1ω7c) (Fierer et al., 2003). The total PLFA of soil microorganisms is the sum of the PLFA of each group of microorganisms.

### 2.6 Statistical analysis

The data for each subplot were expressed as the mean of three soil samples, so data analysis was carried out on the data duplicate value (*n* = 3). And the data analysis was performed according to the following steps. First, one‐way analysis of variance (ANOVA) and least significant difference (LSD) tests were used to examine the differences of soil physicochemical properties and microbial community structure among the different rest‐grazing treatments. And then the independent sample *T*‐tests were used to examine the response of soil physicochemical properties and microbial community structure to fertilization treatment. Second, the correlation analysis was used to test the relationship between soil physicochemical properties and microbial community structure. Finally, the redundancy analysis method (RDA) was used to evaluate the associations between soil physicochemical properties and community microorganisms. The above analysis used Microsoft Excel 2013 to organize and summarize the original data, and used SPSS25.0 statistical software to conduct statistical analysis on the data. Histogram of microbial community compositions and percentage increase of fertilization to the maximum value of all PLFA groups were drawn with Origin 2021. The graphs of correlation analysis were performed using the “ggplot2” and “ggthemes” packages in R 4.2.2 and RDA employed CANOCO 5.0 to complete (Lu et al., 2023). *p* < .05 was considered statistically significant.

## 3 RESULTS

### 3.1 Soil properties

The effects of rest‐grazing and fertilization on soil physicochemical properties and microbial carbon and nitrogen during the regreen‐up period were analyzed. The results showed that: under the treatment with non‐fertilization, SOC and TN in the days of rest‐grazing were significant differences. Specifically, SOC was significantly higher than the other 4 treatments at 20 days of rest‐grazing, and TN was significantly lower than the other 3 treatments at 30 and 50 days of rest‐grazing. Under fertilization treatments, pH was significantly higher than the other 3 treatments (20d, 30d, and 40d) at the CK group. MBN was significantly different in the number of rest‐grazing days, and the specific situation was that the CK group was significantly higher than the other 4 treatments. Other soil physicochemical properties and microbial carbon and nitrogen did not show significant differences between days of rest‐grazing (Table 1). Compared non‐fertilization with fertilization, pH in fertilization treatment was significantly higher than that in non‐fertilization treatment. Other soil physicochemical properties and microbial carbon and nitrogen did not show significant differences between non‐fertilization and fertilization treatment (Table S1).

**TABLE 1:** Variability analysis of soil properties.

Classification	Index	Non‐fertilization		Fertilization	
		F	*p*	F	*p*
Soil physicochemical properties	SM	0.113	.975	0.286	.881
	pH	0.791	.557	4.141	.031*
	SOC	23.331	.001**	1.090	.412
	TN	8.503	.003**	1.342	.320
	NH_4_^+^‐N	1.620	.244	0.498	.738
	NO_3_^−^‐N	0.379	.819	0.574	.688
	TP	0.660	.634	1.023	.441
	AP	0.453	.768	0.076	.988
	TK	0.125	.970	0.965	.468
Soil microbial properties	MBC	0.538	.711	3.464	.051
	MBN	3.156	.064	5.797	.011*

*Note*: *Means significant difference between different days of rest‐grazing under the same treatment, *p < .05, **p < .01.

Abbreviations: AP, available phosphorus; MBC, soil microbial biomass carbon; MBN, soil microbial biomass nitrogen, (*n* = 3); NH_4_^+^−N, ammonium nitrogen; NO_3_^‐^−N, nitrate nitrogen; pH, potential of hydrogen; SM, soil moisture; SOC, soil organic carbon; TK, total potassium; TN, total nitrogen; TP, total phosphorus.

### 3.2 Microbial community compositions

**FIGURE 2:**
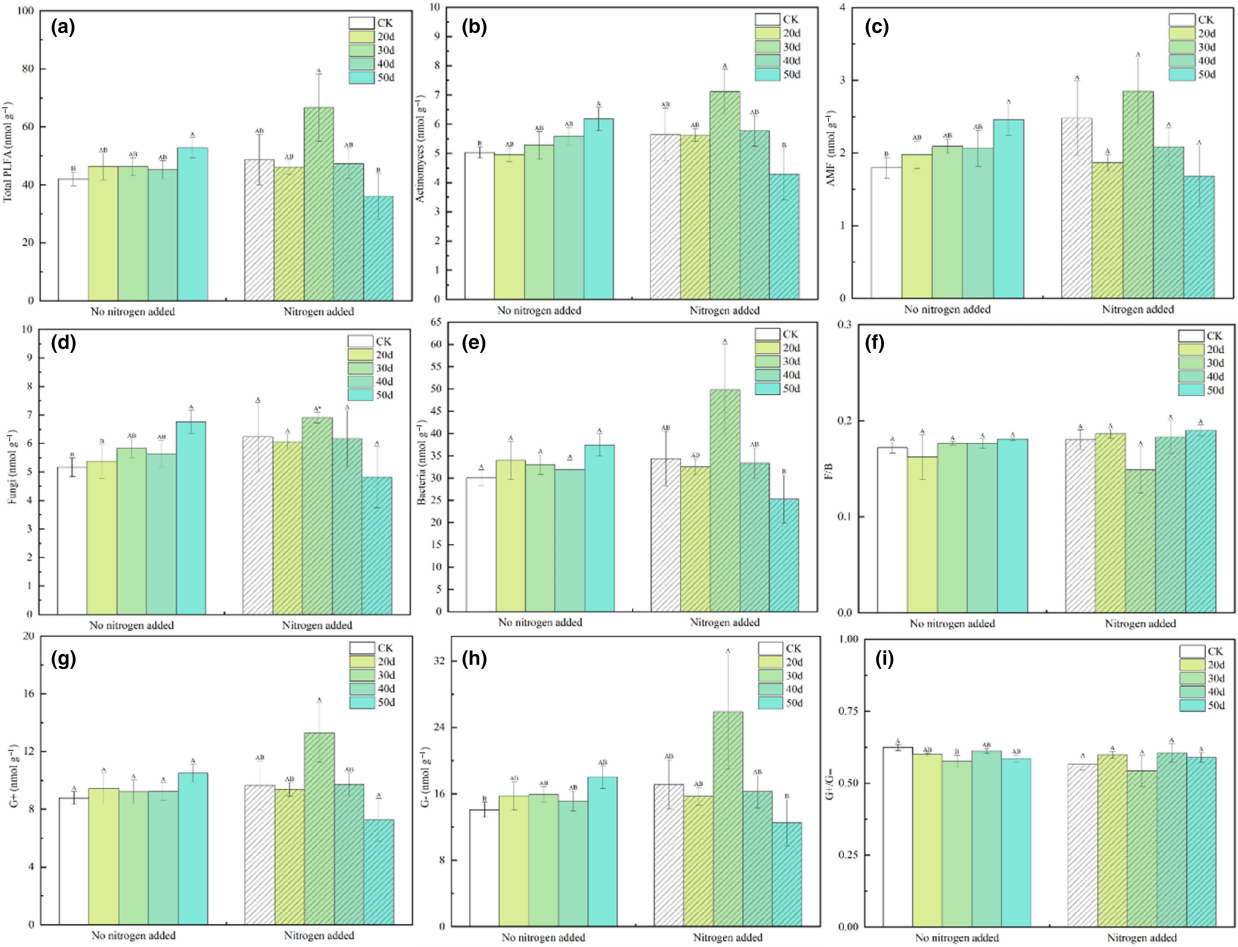
The content of phospholipid fatty acids in soil microorganisms. *Note*: These histograms show the content of phospholipid fatty acids in soil microorganisms under rest‐grazing and fertilization. Different capital letters in the same picture indicate significant differences between different days of rest grazing under the same treatment; * Means significant difference between non‐ fertilization and fertilization under the same number of rest‐grazing days, *, *p* < .05. CK, control check group; 20d, rest‐grazing from June 10 to June 30; 30d, rest‐grazing from May 30 to June 30; 40d, rest‐grazing from May 20 to June 30; 50d, rest‐grazing May 10 to June 30; F/B, the ratio of fungal to bacterial PLFAs; G+, gram‐positive bacterial PLFAs; G−, gram‐negative bacterial PLFAs; G+/G−, the ratio of gram‐positive to gram‐negative bacterial PLFAs, (a), the biomass of total phospholipid fatty; (b), the biomass of actinomyces; (c), the biomass of arbuscular mycorrhizal fungi; (d), the biomass of fungi; (e), the biomass of bacteria ; (f), the ratio of fungi to bacteria; (g), the biomass of gram‐positive bacteria; (h), the biomass of gram‐negative bacteria; (h), the ratio of gram‐positive bacteria to gram‐negative bacteria, (*n* = 3).

**FIGURE 3:**
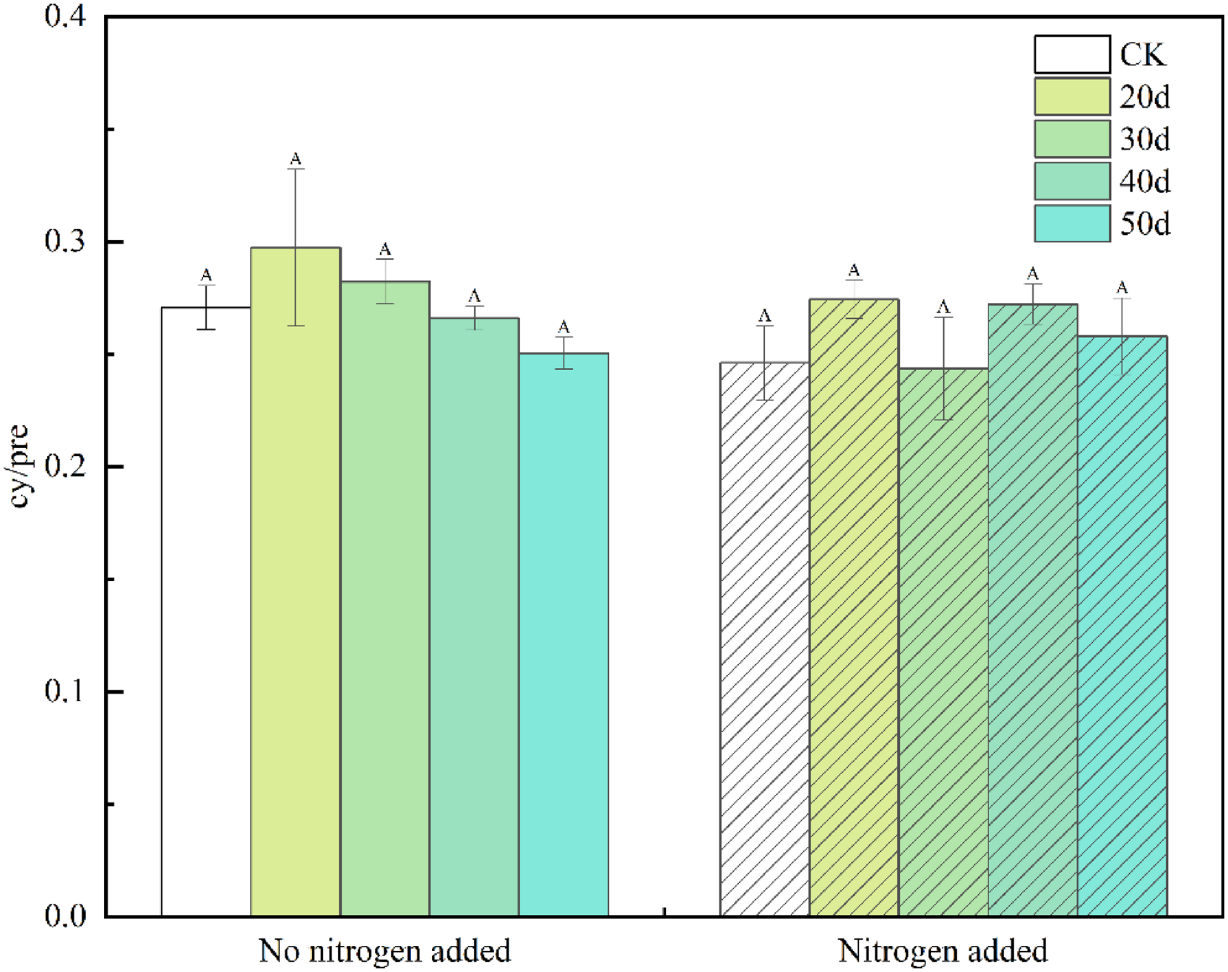
The bacterial stress indicators in soil microorganisms. *Note*: This histogram shows the bacterial stress indicators of (cy‐17:0 + cy‐19:0) to (16:1 ω7c + 18:1ω7c) the content of phospholipid fatty acids in soil microorganisms under rest‐grazing and fertilization. Different capital letters in the same picture indicate significant differences between different days of rest grazing under the same treatment; *p* < .05. CK, control check group; 20d, rest‐grazing from June 10 to June 30; 30d, rest‐grazing from May 30 to June 30; 40d, rest‐grazing from May 20 to June 30; 50d, rest‐grazing May 10 to June 30; cy/pre, ratio of cy‐17:0 + cy‐19:0 to 16:1 ω7c + 18:1ω7c, (*n* = 3).

Statistical analysis of the soil microbial community showed that under non‐fertilization treatment, the four indicators of B, F/B, G+, and G− had no significant difference among rest‐grazing treatments (Figure 2e–h). The three indicators total PLFA, Act, and AMF were significantly higher than CK group at 50 days of rest grazing (*p* = .049, *p* = .036, *p* = .030; Figure 2a–c). Fungi (F) was significantly higher than CK group and 20 days of rest‐grazing at 50 days of rest‐grazing (*p* = .270, *p* = .475; Figure 2d). G+/G− was significantly lower than that of the CK group at 30 days of rest grazing (*p* = .230; Figure 2i). Under the fertilization treatment, the four indexes of AMF, F, F/B, and G+/G− had no significant difference among rest grazing treatments (Figure 2c,d,f,i). Total PLFA, Act, B, G+, and G−: these five indicators were significantly higher at 30 days of rest‐grazing than at 50 days of rest grazing (*p* = .020, *p* = .018, *p* = .018, *p* = .013, *p* = .030; Figure 2a,b,e,g,h). When compared the non‐fertilization treatment with the fertilization treatment, the results showed that F was significantly lower than that of the fertilized treatment (*p* = .045; Figure 2d). Except for the F index, other indicators did not show significant differences between the non‐fertilization and fertilization treatments. The rest‐grazing time did not significantly changed the bacteria stress index, and this effect was not modified by the fertilization treatment (Figure 3).

**FIGURE 4:**
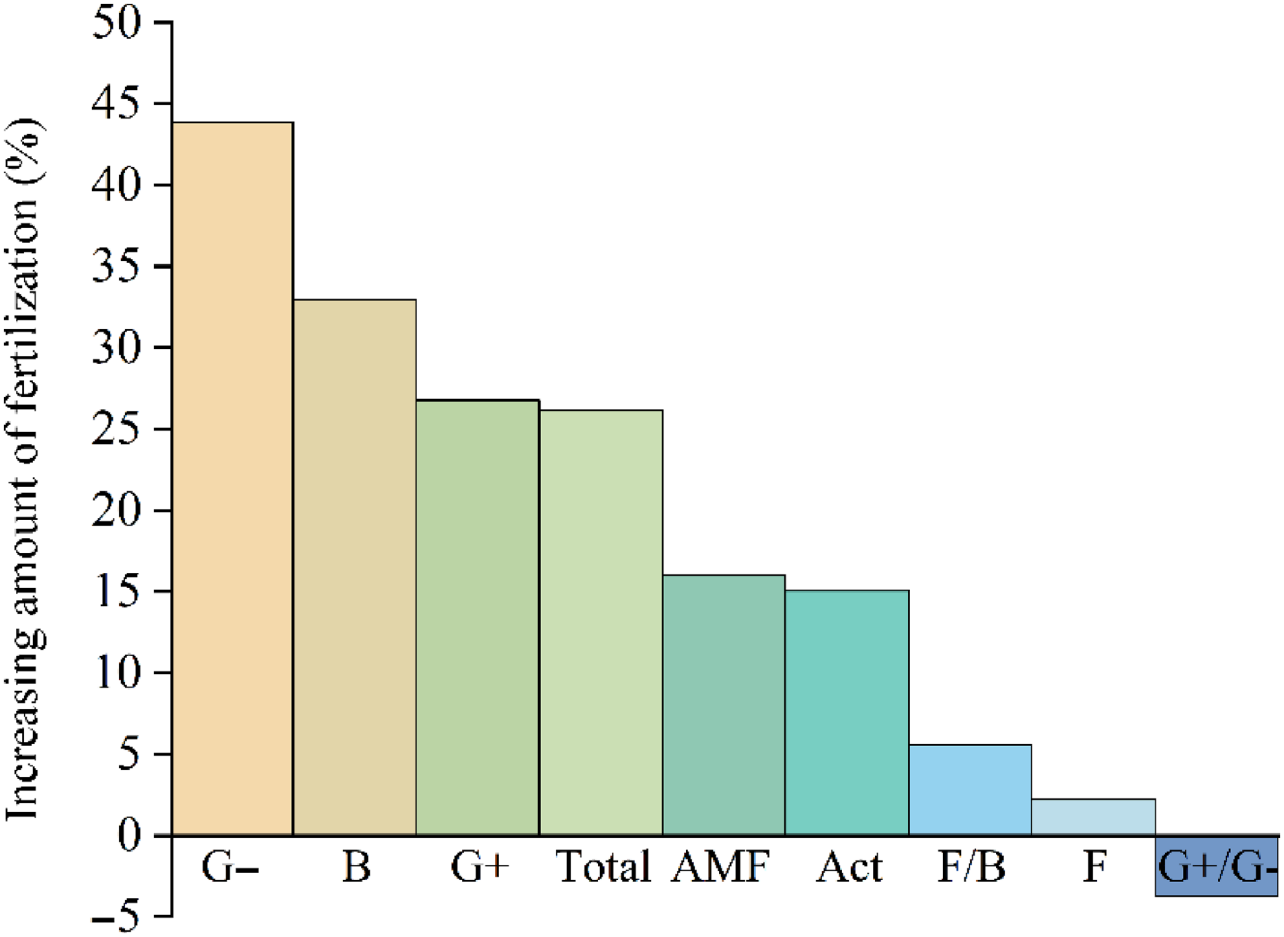
Percentage increase of fertilization to the maximum value of all PLFA groups. *Note*: This histogram shows discrepant values of the maximum value of all PLFA groups between the fertilization and no fertilization treatment. G−, gram‐negative bacterial PLFAs; B, total bacterial PLFAs; G+, gram‐positive bacterial PLFAs, Total, the sum of the PLFA of each group of microorganisms; AMF, arbuscular mycorrhizal fungi PLFAs; Act, actinomyces PLFAs; F/B, the ratio of fungal to bacterial PLFAs; F, fungal PLFAs; G+/G−, the ratio of gram‐positive to gram‐negative bacterial PLFAs, (*n* = 3).

Fertilization has changed the biomass of soil microbial community in different rest‐grazing time, especially enhancing the effects of 30d rest‐grazing and weakening the effects of 50d rest‐grazing (Table S2). In addition, the maximum of all PLFA groups was compared between fertilization and no fertilization. It was shown that fertilization under rest‐grazing could increase the maximum value of all PLFA groups (except the ratio of G+/G−). In detail, the order of the increase of all PLFA groups by fertilization is as follows G−, B, G+, total, AMF, Act, F/B, and F. And they increased by 43.83%, 32.97%, 26.76%, 26.16%, 16.01%, 15.05%, 5.56%, and 2.22%, respectively. Only the ratio of G+/G− decreased by 3.72% (Figure 4).

### 3.3 Relationships between the microbial community compositions and environmental variables

**FIGURE 5:**
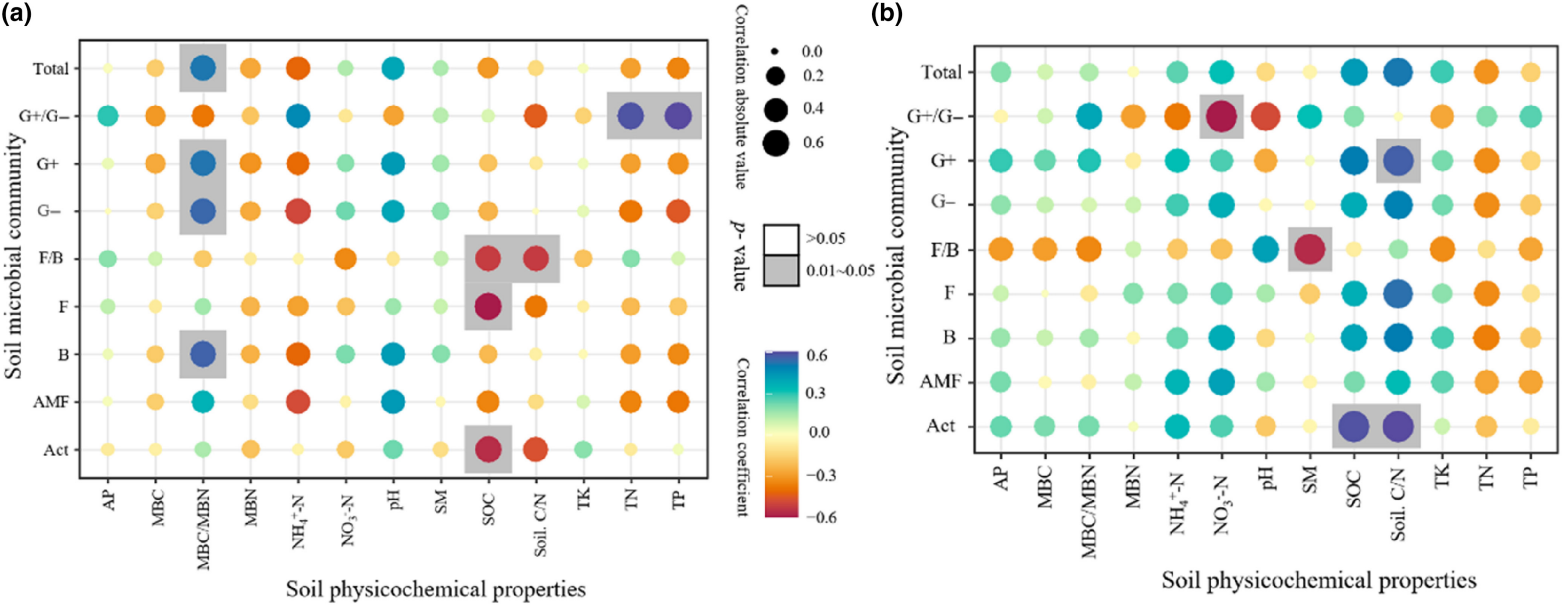
Correlations of the soil physicochemical properties and microbial properties. *Note*: These correlation diagrams show the correlations of the soil physicochemical properties and microbial properties. (a): no fertilization treatment; (b) fertilization treatment; Total, the sum of the PLFA of each group of microorganisms; G+/G−, the ratio of gram‐positive to gram‐negative bacterial PLFAs; G+, gram‐positive bacterial PLFAs; G−, gram‐negative bacterial PLFAs; F/B, the ratio of fungal to bacterial PLFAs; F, fungal PLFAs; B, total bacterial PLFAs; AMF, arbuscular mycorrhizal fungi PLFAs; Act, actinomyces PLFAs; AP, available phosphorus; MBC, soil microbial biomass carbon; MBN, soil microbial biomass nitrogen; NH_4_^+^ −N, ammonium nitrogen; NO_3_^‐^−N, nitrate nitrogen; pH, potential of hydrogen; SM, soil moisture; SOC, soil organic carbon; soil C/N, the ratio of total carbon content to total nitrogen content in soil; TK, total potassium; TN, total nitrogen; TP, total phosphorus, (*n* = 3).

The correlation analysis between soil physicochemical properties and microbial properties showed that: under non‐fertilization treatment, the microbial community is related to soil environmental factors such as MBC/MBN, SOC, soil C/N, TN, and TP. The specific performance is as follows, MBC/MBN was significantly positively correlated with Total PLFA, G+, G−, and B (*r* = .521, *p* = .046; *r* = .525, *p* = .044; *r* = .553, *p* = .032; *r* = .564, *p* = .028). SOC was significantly negatively correlated with F/B, F and A (*r* = −.525, *p* = .044; *r* = −.604, *p* = .017; *r* = −.564, *p* = .028). Soil C/N was significantly negatively correlated with F/B (*r* = −.529, *p* = .043).TN and TP were significantly positively correlated with G/G− (*r* = .593, *p* = .020; *r* = .611, *p* = .016; Figure 5a). Under fertilization treatment, the microbial community is related to soil environmental factors such as NO_3_^−^‐N, SM, SOC, soil C/N. The specific performance is as follows, NO_3_^−^‐N was significantly negatively correlated with G+/G− (*r* = −.582, *p* = .023). SM was significantly negatively correlated with F/B (*r* = −.550, *p* = .034). SOC was significantly positively correlated with A (*r* = .550, *p* = .034). Soil C/N was significantly positively correlated with G+ and A (*r* = .525, *p* = .044; *r* = .564, *p* = .028; Figure 5b).

Through fertilization, MBC/MBN was positively affected with Total PLFA, G+, G−, and B, but there is no significant difference between them. SOC and soil C/N have changed from negative correlation to positive correlation with soil microorganisms. G+/G− has changed from a significant positive correlation with TN and TP to a significant negative correlation with NO_3_^−^‐N. G+ has changed from a significant positive correlation with MBC/MBN to a significant positive correlation with soil C/N. F/B has changed from a significant negative correlation with SOC and soil C/N to a significant negative correlation with SM. Act has changed from a significant negative correlation with SOC to a significant positive correlation with SOC and soil C/N.

**FIGURE 6:**
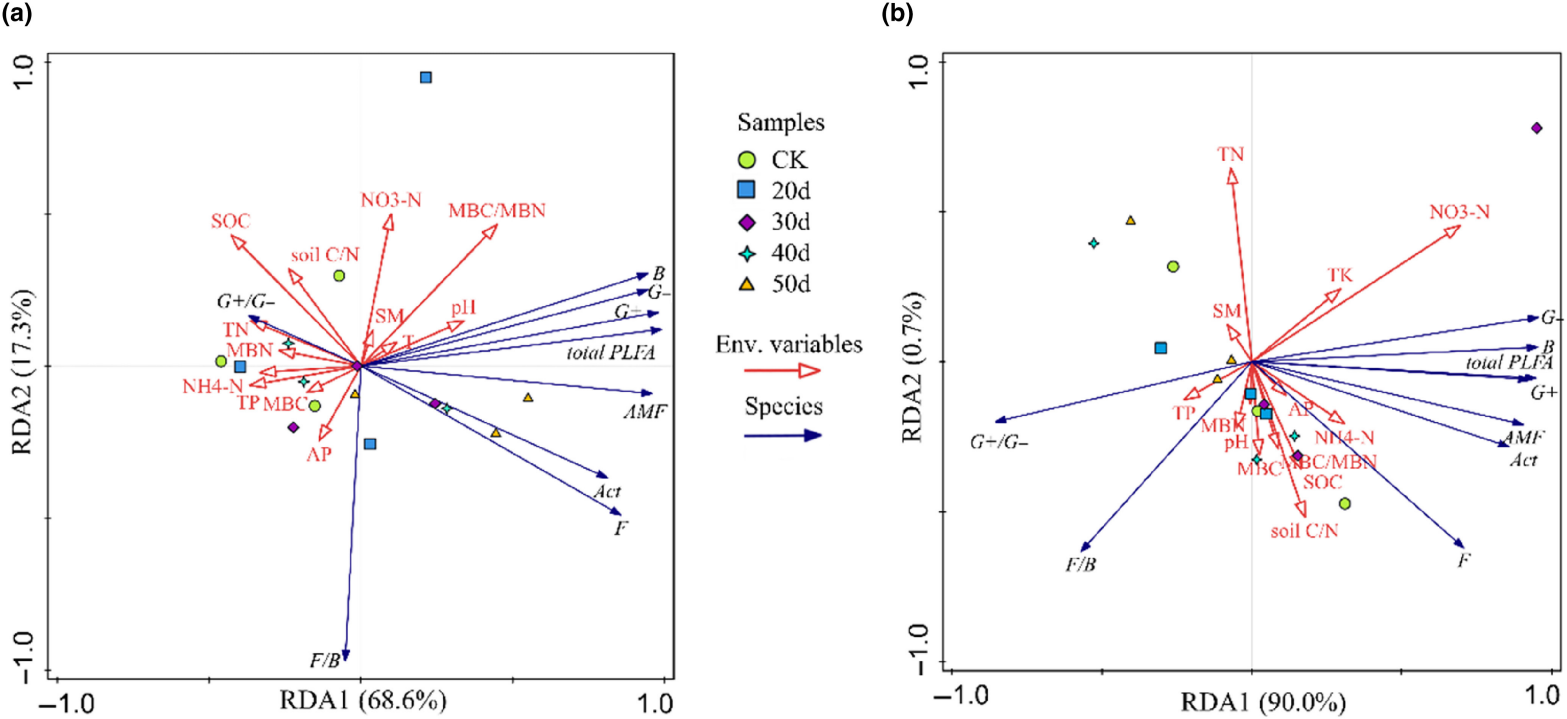
Redundancy analysis of soil physicochemical properties and microbial properties. *Note*: (a): no fertilization treatment; (b) fertilization treatment; CK, control check group; 20d, rest‐grazing from June 10 to June 30; 30d, rest‐grazing from May 30 to June 30; 40d, rest‐grazing from May 20 to June 30; 50d, rest‐grazing May 10 to June 30; Env. Variables, physicochemical properties namely environment variables; Species, all PLFA groups namely species variables; total PLFA, the sum of the PLFA of each group of microorganisms; G+/G−, the ratio of gram‐positive to gram‐negative bacterial PLFAs; G+, gram‐positive bacterial PLFAs; G−, gram‐negative bacterial PLFAs; F/B, the ratio of fungal to bacterial PLFAs; F, fungal PLFAs; B, total bacterial PLFAs; AMF, arbuscular mycorrhizal fungi PLFAs; Act, actinomyces PLFAs; AP, available phosphorus; MBC, soil microbial biomass carbon; MBN, soil microbial biomass nitrogen; NH_4_^+^−N, ammonium nitrogen; NO_3_^‐^−N, nitrate nitrogen; pH, potential of hydrogen; SM, soil moisture; SOC, soil organic carbon; soil C/N, the ratio of total carbon content to total nitrogen content in soil; TK, total potassium; TN, total nitrogen; TP, total phosphorus, (*n* = 3).

Redundancy analysis (RDA) of soil physicochemical properties and microbial properties showed that: when the rest‐grazing is adopted the microbial community is mainly influenced by MBC/MBN, SOC, TP, SOC, soil C/N, MBC, and MBN. But after fertilization, the microbial community is mainly influenced by NO_3_^−^‐N, TN, NH_4_^+^‐N, TK, SOC, and soil C/N. The specific performance is as follows. Under non‐fertilization treatment, soil physicochemical properties explained 85.8% of the total variation in the data, of which the Axes1and 2 explaining 68.6% and 17.3% of the total variation, respectively. MBC/MBN explained 17.9% of the variation (*p* = .052), SOC explained 16.9% of the variation (*p* = .058), TP explained 12.6% of the variation (*p* = .058), soil C/N explained 8.3% of the variation (*p* = .174), MBC explained 7.1% of the variation (*p* = .182), and MBC explained 6.7% of the variation. But the effects of these factors on the microbial community structure were not significant (Figure 6a). Under the fertilization treatment, soil physicochemical properties explained 90.7% of the total variation in the data, of which the Axes1and 2 explaining 90.0% and 0.7% of the total variation, respectively. NO_3_^−^‐N explained 43.5% of the variation and significantly affected the microbial community structure (*p* = .018). In addition, TN explained 9.4% of the variation (*p* = .078); NH_4_^+^‐N explained 7.5% of the variation (*p* = .164); TK explained 6.4% of the variation (*p* = .214); SOC explained 6.3% of the variation (*p* = .190) and soil C/N explained 6.1% of the variation (*p* = .266) but the effects of these factors on the microbial community structure were not significant (Figure 6b).

## 4 DISCUSSION

### 4.1 Effects of rest‐grazing time on soil microbial community

Soil microorganisms play a key role in the circulation and transformation of soil physicochemical properties and nutrients (Sa et al., 2012). Differences in rest‐grazing time during the regreen‐up period usually induced to strong changes in soil physicochemical properties, which in turn affect the quantity and structure of soil microbial communities. Under the non‐fertilization treatment, it was found that total PLFA, Act, AMF and F showed an ever‐increasing biomass with the increase of rest‐grazing time and the highest was at 50 days of rest‐grazing, and they were all significantly higher than CK. This may be related to the growth and development of plants (Wang et al., 2023). When plants enter the mature stage of growth and development, they have a greater demand for soil nutrients and promote the activities of microorganisms in the soil, increasing the content of soil microorganisms PLFA, AMF, and F (Boeddinghaus et al., 2019). This is consistent with studies showing that total PLFA, Act, and F were significantly higher in rest‐grazing compared with free grazing. However, the biomass of other flora did not show significant differences in rest‐grazing time, which may be due to the fact that the rest‐grazing experiment did not show any changes when the period of time was relatively short (Johnson et al., 2008).

The ratio of gram‐positive to gram‐negative bacteria can reflect the level of soil substrate supply, that is, the level of organic matter supply (Siles et al., 2023). There is a negative relationship between the substrate nutrient organic nitrogen and its ratio (G+/G−), that is to say, the more the former, the smaller the ratio, and the higher the nutrient stress to microorganisms (Chen et al., 2023). Studies have shown that G+/G− is the highest in continuous grazing compared with different rest‐grazing treatments (Yang et al., 2022). When continuous grazing is used, the aboveground and underground amount of vegetation decreases, the litter decreases and the nutrient circulation slows down (Qu et al., 2021). In this experiment, SOC was significantly higher than CK at 30 days of rest‐grazing, while G+/G− was significantly lower than CK at 30 days of rest‐grazing. It may be that the soil microorganisms get a good nutrient supply during 30 days of rest‐grazing, which is conducive to the sustainable operation of the entire ecosystem and the stronger the buffering and anti‐interference ability of the entire ecosystem (Lopes & Fernandes, 2020).

### 4.2 Effects of rest‐grazing and fertilization on soil microbial community

Fertilization under rest‐grazing could increase the maximum value of biomass of all PLFA groups. In the fertilization treatment, the concrete changes are as follows, the biomass of each microbial group and microbial total PLFA changed in an “inverted V” shape with the increase of rest‐grazing time, and the value was the largest at 30 days of rest‐grazing, and the smallest at 50 days of rest‐grazing. This shows that moderate rest grazing and fertilization can promote the biomass of each microbial group, thereby increasing the total microbial population. In this experiment, there was no obvious change the biomass of bacterial after fertilization, and there was only a small fluctuation up and down. The research concluded that nitrogen fertilizer is not conducive to the growth of bacteria, but Lovell's research proved that nitrogen fertilization is conducive to the growth of bacteria, and may also directly affect other microbial communities by changing the availability of nutrients (Lovell et al., 1995; Wang & Hou, 2004). Therefore, the effect of nitrogen fertilizer application on bacteria is still controversial, probably because bacteria are more sensitive to the amount of nitrogen fertilizer (Lian et al., 2022). As a result, it is necessary to carry out research on the amount of fertilization (Heinsoo et al., 2020). The ratio of fungi to bacteria is usually used to characterize the changes in the biomass of fungi and bacteria in the soil, the relative abundance of the two populations and the stability of the soil ecosystem (Thiet et al., 2006). In this experiment, the fungi biomass increased compared with no fertilization except for 50 days of rest‐grazing in the fertilization treatment, and the difference was significant at 30 days of rest‐grazing. This is consistent with the results that the research shows that after applying nitrogen fertilizer, the soil fungi biomass has increased, and nitrogen fertilizer has a certain promotion effect on the growth of fungi (Yu et al., 2015).

In the fertilization treatment, the ratio of G+/G− was lower than that of non‐fertilization except for 50 days, which indicated that the addition of nitrogen fertilizer could effectively improve the nutritional status of the soil except for 50 days of rest‐grazing. However, when resting for 50 days, it may be due to the excessively long resting time and the addition of nitrogen fertilizer, livestock trampling and reduced defecation, which may lead to changes in microbial activity and bacterial ratio (Wang, Xin, et al., 2021). This phenomenon is consistent with the research results of the value of G+/G− after adding nitrogen fertilizer to the fence was higher than that of the grazing group (Zhang et al., 2012). But this is only a single feature point, not enough to explain the complex functional characteristics of the ecosystem. Therefore, more indicators are needed for comprehensive research on the response mechanism of ecosystem buffering and resistance to grassland use patterns (Luo et al., 2023). Our finding of no difference of soil bacterial activity with fertilization treatment is not consistent with the research results of the N deposition might decrease bacterial activity (Shao et al., 2018). This phenomenon may be due to the short fertilization time in this study.

Compared the non‐fertilization and fertilization treatments, the results show that the ecosystem stability degree at 20 days with non‐fertilization and rest grazing is lower than other rest‐grazing times, and the ecosystem stability at 30 days when fertilization and rest‐grazing is lower than other rest‐grazing times. In addition, the study found that the ecosystem was the most stable at 50 days of rest‐grazing. From the results of this study, it can be concluded that for the grassland ecosystem, the soil environment stability of the rest‐grazing treatment is higher than that of the grazing treatment (Lang et al., 2021). This was mainly due to the absence of livestock trampling in the rest‐grazing treatment, which preserved the structural integrity of the fungi mycelium and made its biomass grow continuously and steadily, thus increasing the fatty acid ratio of fungi and bacteria (Khatri et al., 2022). This phenomenon is consistent with the research results of Stipa grandis grassland in Inner Mongolia, and the results showed that the fatty acid ratio of fungi and bacteria in the fence treatment was higher than that in the grazing treatment (Zhao et al., 2011).

However, long‐term rest‐grazing and fertilization may lead to changes in the soil environment in which microorganisms survive and lead to a decrease in soil microbial activity (Dos Santos et al., 2022). This may be due to long‐term rest‐grazing and fertilization, changes in aboveground vegetation types, increase in aboveground vegetation litter, plants which need nitrogen‐rich habits, changes in plant root exudates, and changes in soil physicochemical properties (Zong et al., 2021). Changes in soil environment and vegetation may result in reduced microbial biomass (Li, Yang, et al., 2023). Therefore, in the restoration of degraded alpine meadows, rest‐grazing and fertilization measures according to local conditions should be adopted to achieve good ecological effects. Instead of simply performing long‐term rest‐grazing and fertilization on grassland, a combination of moderate rest grazing and fertilization measures should be taken to obtain higher ecological benefits (Blüthgen et al., 2012).

### 4.3 Relationships between soil microbial community characteristics and potential drivers

Characteristics such as the quantity and composition of the soil microbial community can quickly reflect the soil quality, the change of soil physicochemical properties has become the key reason for the change of microbial metabolic characteristics (Ni et al., 2022). According to the correlation analysis between soil physicochemical properties and microbial communities, six soil physical and chemical factors, MBC/MBN, NO_3_^−^‐N, SOC soil C/N, TP, and TN, were screened out to have significant correlations with microbial communities (Figure 4). It may be that although there are many soils environmental factors, they may not all directly act on microorganisms (Glaser et al., 2015). RDA indicated that soil physicochemical properties explained the largest variance in the microbial community structure (Figure 5), with MBC/MBN, NO_3_^−^‐N, SOC, and soil C/N as the most important factors (Table S3). In the non‐fertilization treatment, SOC and soil C/N were negatively correlated with the biomass of all PLFA groups, but in the treatment of fertilization, SOC and soil C/N were positively correlated with the biomass of all PLFA groups. This shows that the amount of soil organic carbon has a direct impact on the number and function of microorganisms (Yang et al., 2021). After applying nitrogen fertilizer, the increase of SOC and soil C/N will increase the biomass of each microbial group and the total PLFA biomass of microorganisms to a certain extent. It may be that the addition of nitrogen fertilizer changed the soil C/N, plants which need nitrogen‐rich habits increased, and their plant root exudates changed, which had a positive impact on the soil microbial community structure (Wang, Li, et al., 2021). In addition, the study found that the MBC/MBN was positively correlated with the biomass of each microbial group and microbial total PLFA. But after fertilization, instead of MBC/MBN, NO_3_^−^‐N not only have a certain influence on the quantity, composition and structure of the microbial community, but also actively regulate the direction of microbial function.

## 5 CONCLUSION

The aim of this study was to investigate the response of soil microbial community structure to rest‐grazing and fertilization during the regreen‐up period. The findings showed that microbial populations in the soil under rest‐grazing were higher than those under grazing, and a longer period of rest‐grazing lead to higher biomass of soil microorganisms. However, fertilization changed the response of microorganisms to rest‐grazing time and lead to an increase in the maximum value of biomass in all PLFA groups. Fertilization also modified the main parameters explaining biomass and composition changes, where NO_3_^−^‐N became one of the primary factors. This study evaluated soil physicochemical properties, microbial community structure, and their correlation with rest‐grazing time and fertilization. The findings underscore the significance of rest‐grazing time and fertilization for microbial communities and provide essential data for managing degraded alpine meadows.

## AUTHOR CONTRIBUTIONS

**Xuanbo Zhou:** Data curation (equal); investigation (equal); software (lead); writing – original draft (lead). **Xiaoli Wang:** Data curation (equal); funding acquisition (lead); resources (lead); writing – review and editing (lead). **Yushou Ma:** Methodology (lead); project administration (lead); supervision (lead); writing – review and editing (equal). **Yanlong Wang:** Investigation (lead); resources (equal); writing – review and editing (supporting). **Yuan Ma:** Data curation (supporting); investigation (supporting); writing – review and editing (supporting). **Lele Xie:** Data curation (supporting); investigation (supporting); writing – review and editing (supporting).

## CONFLICT OF INTERST STATEMENT STATEMENT

The authors declare no competing interests.

## Supporting information


Table S1‐S3.

## Data Availability

Experimental data have been archived in Data Dryad: https://datadryad.org/stash/share/BhqV4l9j0WnnJudOKye87Zb5baJ3PkDHkQssqxhPR4g. The data that support the findings of this study are openly available in DRYAD at 10.5061/dryad.280gb5mv6.
