# Supplementary material for: Fertilization can accelerate the pace of soil microbial community response to rest‐grazing duration in the three‐river source region of China
Source: Ecol Evol. 2023 Nov 27;13(11):e10734. doi: 10.1002/ece3.10734 (PMC10680436; doi:10.1002/ece3.10734)
Supplement: Supplementary file 1 — Table S1‐S3. [file ECE3-13-e10734-s001.docx]

**Supporting Information**

Additional Supporting Information may be found in the online version of this article:

**Appendix S1.** Tables.

**Table S1.** The independent sample T-test between non-fertilization and fertilization.

**Table S2.** Effect sizes of fertilization on microbial properties.

**Table S3.** Redundancy analysis of soil physicochemical properties and microbial properties using forward selection with a Monte Carlo permutation test

**Appendix S1. Tables.**

**Table S1.** The independent sample T-test between non-fertilization and fertilization

| Soil  physicochemical | *P*-value | | | | | | | | | | |
| --- | --- | --- | --- | --- | --- | --- | --- | --- | --- | --- | --- |
|  | SM | pH | SOC | TN | NH_4_^+^-N | NO_3_^-^-N | TP | AP | TK | MBC | MBN |
| CK | 0.998 | 0.013* | 0.150 | 0.109 | 0.145 | 0.898 | 0.705 | 0.208 | 0.188 | 0.098 | 0.056 |
| 20d | 0.481 | 0.863 | 0.873 | 0.054 | 0.825 | 0.613 | 0.597 | 0.592 | 0.542 | 0.200 | 0.614 |
| 30d | 0.675 | 0.301 | 0.430 | 0.454 | 0.652 | 0.821 | 0.336 | 0.556 | 0.528 | 0.096 | 0.338 |
| 40d | 0.854 | 0.687 | 0.203 | 0.099 | 0.177 | 0.547 | 0.789 | 0.390 | 0.229 | 0.691 | 0.082 |
| 50d | 0.516 | 0.962 | 0.219 | 0.263 | 0.487 | 0.268 | 0.655 | 0.450 | 0.082 | 0.278 | 0.915 |

Note: * means significant difference between non-fertilized and fertilized under the same days of rest-grazing, *, P<0.05. CK: control check group; 20d: rest-grazing from June10th to June 30th; 30d: rest-grazing from May 30th to June 30th; 40d: rest-grazing from May 20th to June 30th; 50d: rest-grazing May 10th to June 30th; SM: soil moisture; pH: potential of hydrogen; SOC: soil organic carbon; TN: total nitrogen; NH_4_^+^−N: ammonium nitrogen; NO_3_^-^—N: nitrate nitrogen; TP: total phosphorus; AP: available phosphorus; TK: total potassium; MBC: soil microbial biomass carbon; MBN: soil microbial biomass nitrogen, (n=3).

**Table S2.** Effect sizes of fertilization on microbial properties

| Microbial community | Fertilization Effect Size | | | | | | | | |
| --- | --- | --- | --- | --- | --- | --- | --- | --- | --- |
|  | total | Act | AMF | F | B | F/B | G+ | G- | G+/G- |
| CK | 0.29 | 0.26 | 0.46 | 0.33 | 0.26 | 0.27 | 0.20 | 0.37 | -0.74 |
| 20d | -0.01 | 0.65 | -0.20 | 0.40 | -0.12 | -0.08 | -0.02 | -0.01 | -0.04 |
| 30d | 0.57 | 0.64 | 0.55 | 0.76 | 0.54 | -0.42 | 0.60 | 0.50 | -0.23 |
| 40d | 0.14 | 0.12 | 0.03 | 0.19 | 0.40 | 0.15 | 0.20 | 0.20 | 0.29 |
| 50d | -0.63 | -0.63 | -0.56 | -0.57 | -0.64 | 0.56 | -0.63 | -0.59 | 0.09 |

Note: CK: control check group; 20d: rest-grazing from June10th to June 30th; 30d: rest-grazing from May 30th to June 30th; 40d: rest-grazing from May 20th to June 30th; 50d: rest-grazing May 10th to June 30th; total: the sum of the PLFA of each group of microorganisms;

Act: actinomyces PLFAs; AMF: arbuscular mycorrhizal fungi PLFAs; F: fungal PLFAs; B: total bacterial PLFAs; F/B: the ratio of fungal to bacterial PLFAs; G+: gram-positive bacterial PLFAs; G−: gram-negative bacterial PLFAs; G+/G−: the ratio of gram-positive to gram-negative bacterial PLFAs, (n=3).

**Table S3.** Redundancy analysis of soil physicochemical properties and microbial properties

using forward selection with a Monte Carlo permutation test

| Treatment | Variables | Explains  (%) | Contribution  (%) | F-ratio | P-Value | Axis | 1 | 2 |
| --- | --- | --- | --- | --- | --- | --- | --- | --- |
| Non- fertilization treatment | MBC/MBN | 17.9 | 18.1 | 2.8 | 0.052 | Eigenvalues | 0.686 | 0.173 |
|  | SOC | 16.9 | 17.1 | 3.1 | 0.058 | Explained variation | 68.570 | 85.820 |
|  | TP | 12.6 | 12.7 | 2.6 | 0.058 | Pseudo-canonical correlation | 0.999 | 0.975 |
|  | soil C/N | 8.3 | 8.4 | 1.9 | 0.174 | Explained fitted variation | 69.440 | 86.910 |
|  | MBC | 7.1 | 7.1 | 1.7 | 0.182 |  |  |  |
|  | MBN | 6.7 | 6.8 | 1.8 | 0.176 |  |  |  |
| Fertilization treatment | NO_3_^-^-N | 43.5 | 47.9 | 10.0 | 0.018 | Eigenvalues | 0.900 | 0.007 |
|  | TN | 9.4 | 10.4 | 4.6 | 0.078 | Explained variation | 89.970 | 90.680 |
|  | NH_4_^+^-N | 7.5 | 8.2 | 2.2 | 0.164 | Pseudo-canonical correlation | 0.953 | 0.974 |
|  | TK | 6.4 | 7.0 | 1.7 | 0.214 | Explained fitted variation | 99.110 | 99.900 |
|  | SOC | 6.3 | 7.0 | 2.0 | 0.190 |  |  |  |
|  | soil C/N | 6.1 | 6.7 | 1.4 | 0.266 |  |  |  |

Note: MBC/MBN: the ratio of soil microbial biomass carbon to soil microbial biomass nitrogen; SOC: soil organic carbon; TP: total phosphorus; soil C/N: the ratio of total carbon content to total nitrogen content in soil; MBC: soil microbial biomass carbon; MBN: soil microbial biomass nitrogen; NO_3_^-^-N: nitrate nitrogen; TN: total nitrogen; NH_4_^+^−N: ammonium nitrogen; TK: total potassium, (n=3).
